# Purifying selection does not drive signatures of convergent local adaptation of lodgepole pine and interior spruce

**DOI:** 10.1186/s12862-019-1438-8

**Published:** 2019-05-28

**Authors:** Mengmeng Lu, Kathryn A. Hodgins, Jon C. Degner, Sam Yeaman

**Affiliations:** 1 Department of Biological Sciences, University of Calgary, 507 Campus Drive NW, Calgary, T2N 4S8 Canada; 2 School of Biological Sciences, Monash University – Clayton Campus, Building 17, Wellington Road, Melbourne, 3800 Australia; 3 Department of Forest and Conservation Sciences, Forest Sciences Centre 3041, University of British Columbia, 2424 Main Mall, Vancouver, V6T 1Z4 Canada

**Keywords:** Clines, Nucleotide diversity, Tajima’s D, Genetic admixture, Hybridization, SNP

## Abstract

**Background:**

Lodgepole pine (*Pinus contorta*) and interior spruce (*Picea glauca*, *Picea engelmannii*, and their hybrids) are distantly related conifer species. Previous studies identified 47 genes containing variants associated with environmental variables in both species, providing evidence of convergent local adaptation. However, if the intensity of purifying selection varies with the environment, clines in nucleotide diversity could evolve through linked (background) selection that would yield allele frequency-environment signatures resembling local adaptation. If similar geographic patterns in the strength of purifying selection occur in these species, this could result in the convergent signatures of local adaptation, especially if the landscape of recombination is conserved. In the present study, we investigated whether spatially/environmentally varying purifying selection could give rise to the convergent signatures of local adaptation that had previously reported.

**Results:**

We analyzed 86 lodgepole pine and 50 interior spruce natural populations spanning heterogeneous environments in western Canada where previous analyses had found signatures of convergent local adaptation. We estimated nucleotide diversity and Tajima’s D for each gene within each population and calculated the strength of correlations between nucleotide diversity and environmental variables. Overall, these estimates in the genes with previously identified convergent local adaptation signatures had no similar pattern between pine and spruce. Clines in nucleotide diversity along environmental variables were found for interior spruce, but not for lodgepole pine. In spruce, genes with convergent adaption signatures showed a higher strength of correlations than genes without convergent adaption signatures, but there was no such disparity in pine, which suggests the pattern in spruce may have arisen due to a combination of selection and hybridization.

**Conclusions:**

The results rule out purifying/background selection as a driver of convergent local adaption signatures in lodgepole pine and interior spruce.

**Electronic supplementary material:**

The online version of this article (10.1186/s12862-019-1438-8) contains supplementary material, which is available to authorized users.

## Background

Lodgepole pine (*Pinus contorta*) and interior spruce (*Picea glauca*, *Picea engelmannii*, and their hybrids) are among the most economically and ecologically valuable forest tree species in western Canada, inhabiting similar environmental gradients across boreal and montane environments. These two species diverged 140–190 million years ago [1]. They exhibit genomic divergence to spatially variable natural selection, and genomic signatures of local adaptation were identified [2]. However, a suite of 47 orthologous genes containing variants associated with cold hardiness and winter temperature variables suggest local adaptation has been convergent at the genome scale in these two distantly related species [2].

Different evolutionary forces, including positive selection, purifying selection and demographic processes, can drive patterns of genetic diversity in conifer species [3–5]. In the present study, we explore the possibility that purifying selection drives the patterns of association between allele frequency and environment that were originally inferred to be convergent local adaptation (in [2]). If the rate of removal of deleterious alleles and their linked alleles (purifying and background selection) varies spatially with environment, clines in nucleotide diversity could evolve that would yield allele frequency-environment signatures resembling local adaptation [6–8]. Purifying selection impacts nucleotide diversity patterns in conifers [9]. Hodgins, Yeaman et al. [10] analyzed the RNAseq data of lodgepole pine and interior spruce and found low expression genes had a large fraction of neutral sites, suggesting purifying selection played a key role in determining evolutionary rate in expressed genes. Additionally, purifying selection effect differed between coding-regions (CDS) and non-CDS [11]. Because regions of low recombination tend to amplify the signature of background selection across greater regions of the chromosome [12, 13], any such clines in nucleotide diversity are predicted to be more extreme in areas of reduced recombination. The similar genome organization between pine and spruce [14] would increase the likelihood that similar signatures of spatially or environmentally varying background selection could be found in both species, and such patterns could have been mistakenly interpreted as convergent local adaptation by Yeaman, Hodgins et al. [2]. Other studies have reported highly similar patterns of nucleotide diversity at the genome scale between distantly related species, along with broad correlations with recombination rate [13, 15], so this provides a plausible alternative explanation for the signatures of convergence observed in these conifers.

In addition to the above possibility, there could also be confounding patterns due to gene flow between species or varieties with differing levels of genetic variation. Interior spruce exists as an advanced-generation hybrid complex across much of western Canada [16], and the parental species appear to differ in levels of standing genetic variation, with Engelmann spruce generally showing higher diversity than white spruce [17]. Lodgepole pine, although showing substantially less genetic structure than interior spruce [2], contains several genetically- and morphologically-distinct varieties [18], including the varieties *contorta* and *latifolia* which are present in the study area. Within the species, *Pinus contorta var. contorta* may have higher levels of genetic diversity than var. *latifolia* [19]. Lodgepole pine is also known to hybridize extensively with jack pine (*Pinus banksiana*) in Alberta and the Northwest Territories [20–23], and genetic diversity has been noted to be lower in jack pine than lodgepole pine [24]. As demography can be a strong driver of nucleotide diversity, it is also important to explore this potential factor when considering the importance of purifying and background selection.

**Fig. 1:**
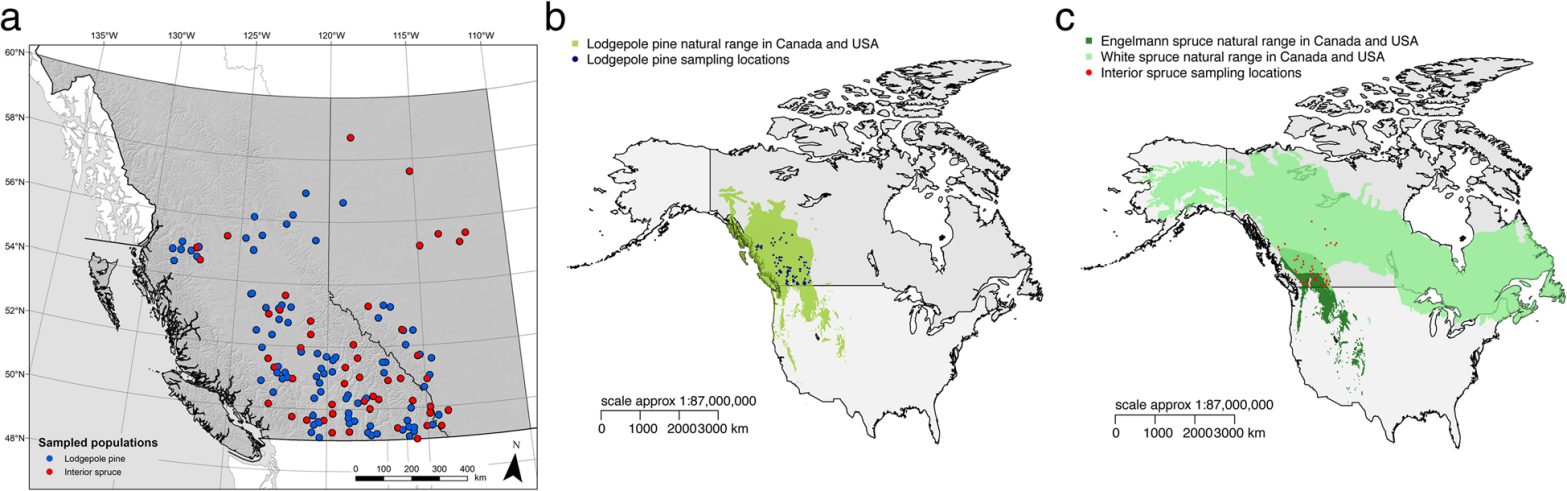
Distribution of lodgepole pine and interior spruce populations analyzed in this study. Blue dots represent the locations of sampled lodgepole pine populations. Red dots represent the locations of sampled interior spruce populations. **a** Sampling locations; **b** Natural range of lodgepole pine and sampling locations; **c** Natural range of interior spruce and sampling locations

In the present study, we tested the possibility that spatially/environmentally varying purifying selection has driven signatures of convergent local adaptation in pine and spruce. We calculated nucleotide diversity for each gene within pine and spruce populations that were previously studied [2] (Fig. 1) and examined the correlation between within-population nucleotide diversity and population environment. We focused on five environmental variables that were most strongly associated with phenotypic adaptation and subsequently used to detect signatures of convergent adaptation. We aimed to answer two questions: (1) Are different patterns of correlation found for genes showing convergent adaptation signatures, compared with top candidate genes not showing these signatures and genes showing no associations (background genes)? (2) Is the pattern of correlation consistent in the 47 genes with signatures of convergent adaptation between pine and spruce? If spatially/environmentally variable purifying selection was driving the previously reported convergent signatures, then we would expect patterns in these genes to be substantially different from the rest of the genes in the genome (question 1). If purifying selection was causing convergence, we would also expect that any spatial/environmental clines in nucleotide diversity would be similar in both species (question 2). We do note that both of these predictions could be realized under either spatial variation in the strength of purifying selection or localized hard selective sweeps and/or stable local adaptation. Thus, it would not be particularly informative if we were to observe similar patterns in pine and spruce at the 47 genes that are substantially different from the genomic background. However, observing either different patterns in each species or lack of difference between the 47 convergent genes and the genomic background would suggest that purifying selection is an unlikely explanation (and thus, convergent local adaptation would remain the most parsimonious interpretation). We also analyzed the genetic admixture structures within pine and spruce populations to examine the extent of hybridization and their potential influence on nucleotide diversity in the studied populations. We aim to obtain insights into potential patterns of purifying selection within lodgepole pine and interior spruce and thereby improve our understanding of the previously-reported signatures of convergent local adaptation in these two species.

## Results

**Fig. 2:**
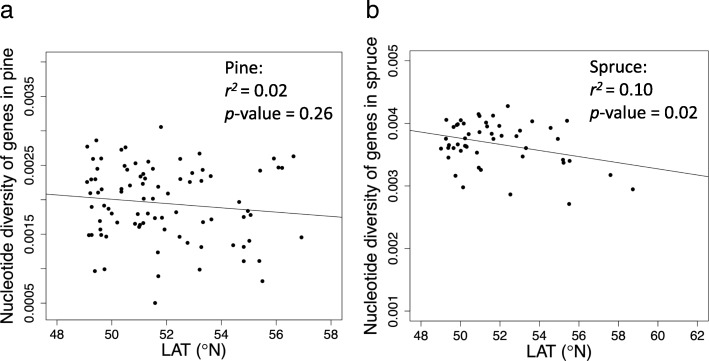
Clinal variation in nucleotide diversity across all genes in the genomes of pine (**a**) and spruce (**b**) (LAT: latitude)

**Fig. 3:**
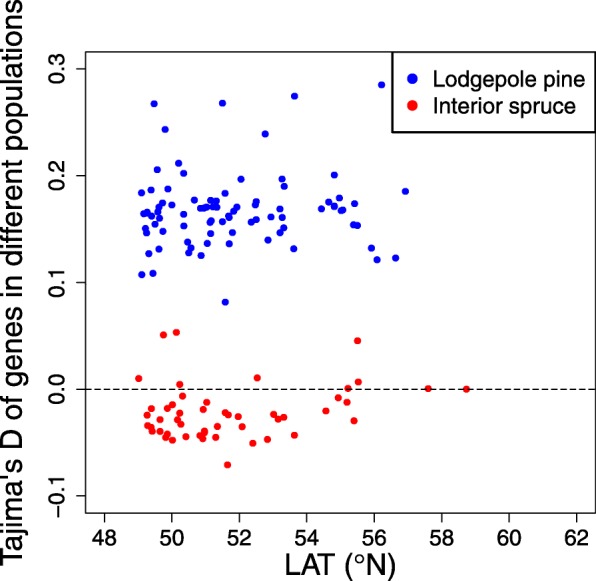
Mean Tajima’s D values across all genes for lodgepole pine and interior spruce populations. Blue dots represent the mean Tajima’s D values across all genes for each lodgepole pine population. Red dots represent the Tajima’s D values across all genes for each interior spruce population (LAT: latitude)

To examine genome-wide patterns as an approximate gauge of the effects of demography on nucleotide diversity, we calculated the strength of association between within-population nucleotide diversity and latitudes across all genes, and found differences when comparing pine and spruce. Pine genes did not show an overall pattern of clinal variation (*r*^2^ = 0.02, *p*-value = 0.26; Fig. 2a), while spruce genes showed a weak but significant clinal variation (*r*^2^ = 0.10, *p*-value = 0.02; Fig. 2b). We observed similar patterns in both coding-regions (CDS) and non-CDS of genes: no clinal variation in pine (Additional file 1: Figure S1a & c); a weak but significant clinal variation in spruce (Additional file 1: Figure S1b & d). We observed distinct differences in Tajima’s D values between pine and spruce (Fig. 3). All the pine populations had positive Tajima’s D values, while most spruce populations had negative or near- zero Tajima’s D values.

**Fig. 4:**
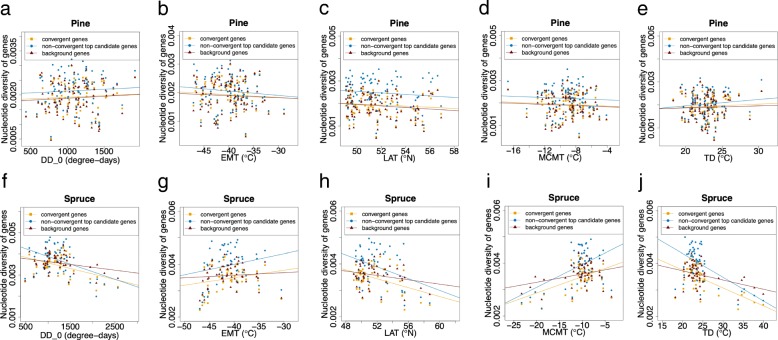
Relationships between environmental variables and nucleotide diversity of genes in lodgepole pine and interior spruce for the convergent genes, non-convergent top candidate genes and background genes. Panels show the comparison of relationships between nucleotide diversity and: **a**, **f** degree-days below 0 °C (DD_0); **b**, **g** 30-year extreme minimum temperature (EMT); **c**, **h** latitude (LAT); **d**, **i** mean temperature of the coldest-month (MCMT); **e**, **j** temperature difference between the warmest and coldest months (TD)

To test our main hypothesis, we then compared the strength of correlation between within-population nucleotide diversity and environmental variables across different types of genes and between species. Pine and spruce showed different patterns when regressing nucleotide diversity of convergent genes, non-convergent top candidate genes and background genes on each environmental variable separately (Fig. 4, Additional file 1: Figures S2 & S3). In pine, we did not observe a significant relationship between any environmental variable and nucleotide diversity across different genic regions (Additional file 1: Table S1). On the contrary, in spruce, environmental variables degree-days below 0 °C, latitude, mean temperature of the coldest month, and temperature difference between the warmest and coldest months (DD_0, LAT, MCMT and TD, respectively) all had significant relationships with nucleotide diversity across different genic regions (Additional file 1: Table S1). Nucleotide diversity tended to be lower in northern or colder areas of the species range than in southern or warmer areas of the range. Thirty-year extreme minimum temperature (EMT) had a weak relationship with nucleotide diversity across convergent genes and non-convergent top candidate genes in spruce, but was not associated with nucleotide diversity of background genes (Additional file 1: Table S1).

**Fig. 5:**
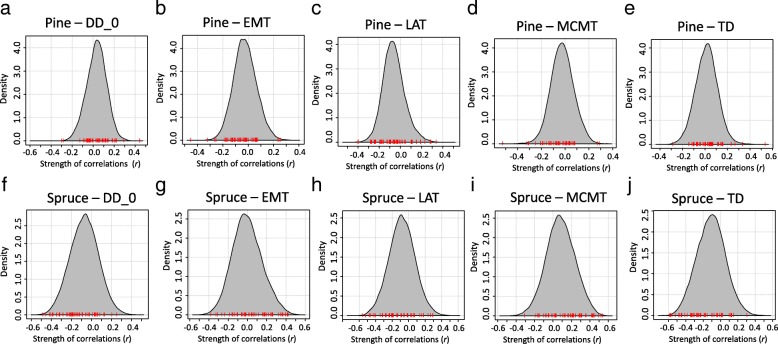
Distribution of strength of correlations (*r*) between environmental variables and nucleotide diversity of genes. Grey polygons show the density of *r* values of all genes. Red bars on the x-axis show the *r* values of convergent genes. Panels show distribution of *r* values for correlations between nucleotide diversity and: **a**, **f** degree-days below 0 °C (DD_0); **b**, **g** 30-year extreme minimum temperature (EMT); **c**, **h** latitude (LAT); **d**, **i** mean temperature of the coldest-month (MCMT); **e**, **j** temperature difference between the warmest and coldest months (TD)

Strength of correlations (*r*) between nucleotide diversity and environmental variables showed different patterns when comparing pine and spruce across different genic regions (Fig. 5, Additional file 1: Figures S4 & S5). t-tests did not show difference in means of *r* values between convergent and non-convergent genes in pine (Table 1). On the contrary, means of *r* values in spruce showed distinct difference between convergent and non-convergent genes, where the *r* values tended to be stronger in convergent genes than in non-convergent genes. However, the difference was less distinct with EMT or LAT than with other environmental variables. The *r* values in non-CDS differed more distinctly than in CDS. Overall, when comparing the *r* values for individual convergent genes between pine and spruce, we found a negative relationship that was significant in all variables except LAT (Table 2, Additional file 1: Figures S6 - S8).

**Table 1:** *t*-tests on strength of correlations (*r*) between nucleotide diversity and environmental variables

Environmental variables^a^	Genic regions^b^	*t*-tests for pine			t-tests for spruce		
		Means of *r* on convergent genes	Means of *r* on non-convergent genes	*p*-values	Means of *r* on convergent genes	Means of *r* on non-convergent genes	*p*-values
DD_0 (degree-days)	Gene	0.044	0.032	0.527	− 0.182	− 0.093	0.010
	CDS	0.028	0.029	0.952	−0.159	− 0.083	0.030
	Non-CDS	0.035	0.031	0.823	−0.193	− 0.089	0.003
EMT (°C)	Gene	−0.054	−0.024	0.108	0.075	0.025	0.165
	CDS	−0.034	−0.022	0.527	−0.113	0.026	0.213
	Non-CDS	−0.040	−0.024	0.342	0.084	0.025	0.094
LAT (∘N)	Gene	−0.058	−0.063	0.856	−0.152	− 0.077	0.051
	CDS	−0.046	−0.055	0.701	−0.130	− 0.068	0.106
	Non-CDS	−0.047	− 0.061	0.522	− 0.164	− 0.075	0.020
MCMT (°C)	Gene	−0.047	−0.024	0.257	0.201	0.103	0.010
	CDS	−0.030	−0.021	0.669	0.172	0.091	0.034
	Non-CDS	−0.037	− 0.023	0.478	0.212	0.100	0.004
TD (°C)	Gene	0.044	0.014	0.121	−0.249	− 0.132	0.002
	CDS	0.033	0.012	0.278	−0.210	− 0.113	0.012
	Non-CDS	0.039	0.015	0.171	−0.257	−0.127	0.001

^a^*DD_0* degree-days below 0 °C, *EMT* 30-year extreme minimum temperature, *LAT* latitude, *MCMT* mean temperature of the coldest month, *TD* temperature difference between the warmest and coldest months. ^b^*CDS* coding-regions, *non-CDS* non-coding-regions

**Table 2:** Correlations of strength of correlations (*r*) between pine and spruce

Environmental variables^a^					
Genic regions^b^	DD_0	EMT	LAT	MCMT	TD
Gene	−0.30 (0.04)	− 0.32 (0.03)	−0.18 (0.22)	− 0.33 (0.02)	−0.34 (0.02)
CDS	−0.42 (0.00)	−0.37 (0.01)	− 0.17 (0.24)	−0.43 (0.00)	− 0.37 (0.01)
Non-CDS	−0.32 (0.03)	− 0.35 (0.01)	−0.13 (0.37)	− 0.35 (0.02)	−0.33 (0.02)

The strength of correlations (*r*) between environmental variables and nucleotide diversity of convergent genes were correlated between pine and spruce. The values of correlation coefficients are followed by *p*-values within parentheses. ^a^*DD_0* degree-days below 0 °C, *EMT* 30-year extreme minimum temperature, *LAT* latitude, *MCMT* mean temperature of coldest-month, *TD* temperature difference between the warmest and coldest months. ^b^*CDS* coding-regions, *non-CDS* non-coding-regions

**Fig. 6:**
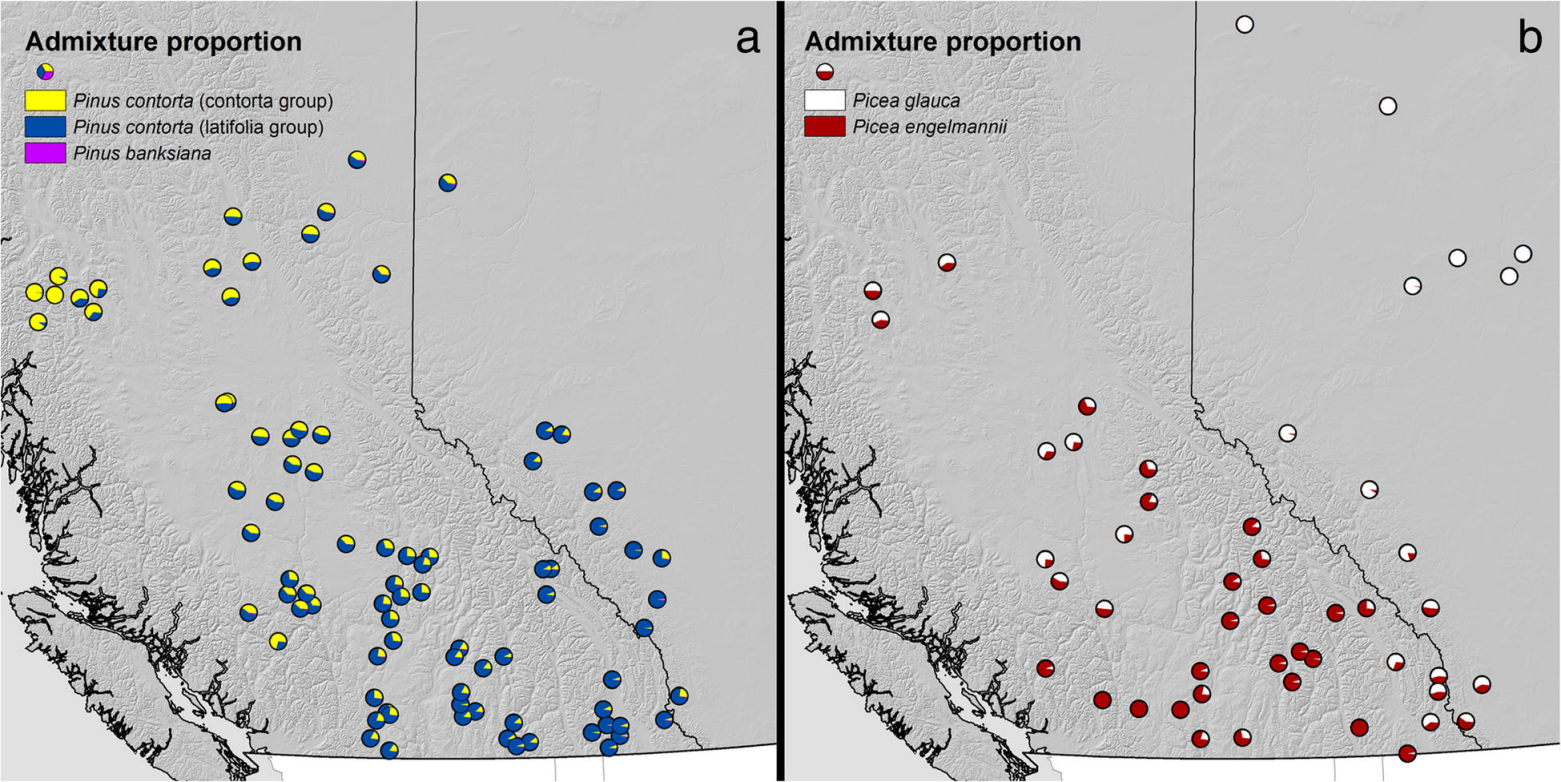
Admixture analyses for studied lodgepole pine (**a**) and interior spruce (**b**) populations. **a** Admixture pie charts for each studied lodgepole pine population. Yellow represents a cluster roughly corresponding to *Pinus contorta var. contorta*. Blue represents a cluster roughly corresponding to *Pinus contorta var. latifolia*. Pink represents *Pinus banksiana*. **b** Admixture pie charts for each studied interior spruce population. Red represents *Picea engelmannii*. White represents *Picea glauca*

Admixture analyses of lodgepole pine populations showed no appreciable hybridization with jack pine in the dataset, with the northernmost populations showing 2–5% jack pine ancestry, and a northwest-southeast gradient of genetic structure within lodgepole pine (Fig. 6a). These clusters align somewhat with expectations for ancestry from *Pinus contorta var. contorta* in the northwest, and *Pinus contorta var. latifolia* moving eastward, although the intermediate ancestry for the majority of populations was not expected and as such this genetic structure does not well-reflect phenotypic divergence between these varieties [18]. The admixture analyses of interior spruce showed a latitudinal gradient of hybridization among the studied populations (Fig. 6b). Most spruce populations in the south of the studied range showed ancestry favouring Engelmann spruce, while populations located in the north of the studied range, especially in the northeastern extent, were primarily white spruce.

## Discussion

We identified clinal patterns of nucleotide diversity in the convergent genes in interior spruce but not in lodgepole pine. If the previously reported signatures of convergent local adaptation were instead driven by spatially/environmentally varying purifying selection, then we should have observed similar clinal patterns in both pine and spruce. In the present study, the lack of similar clinal pattern therefore does not support the hypothesis that spatially/environmentally varying purifying/background selection caused the apparent convergent local adaptation signatures in these two species. In spruce, genes with convergent adaption signatures showed a higher strength of correlations between environmental variables and nucleotide diversity than genes without signatures of convergent adaption, but there was no such disparity in pine (Fig. 5, Table 1, Additional file 1: Figures S4 & S5). We also did not find consistent patterns when comparing the strength of correlations among the 47 convergent genes between pine and spruce (Table 2, Additional file 1: Figures S6 – S8). If anything, these results are somewhat perplexing, as we observe a slight negative correlation overall between the clinal associations in spruce and those in pine: genes with a positive correlation between a given environmental variable and nucleotide diversity in pine tend to have a negative correlation with the same variable and nucleotide diversity in spruce, and vice versa. However, this result is driven largely by a few genes (Additional file 1: Figures S6 – S8), perhaps due to some interaction between species demography and divergent selection that we assume was driving the signatures of convergence, with most genes showing very little significant correlation in either species. Taken together, these results suggest that, if anything, the intensity of purifying selection may vary with environment in spruce, but not in pine. The result also precludes the possibility that localized hard selective sweeps lead to the convergence, otherwise we should have seen similar clines in nucleotide diversity in the convergent genes. Other ecological and/or demographic conditions could also explain the observed patterns in nucleotide diversity, so this study should not be regarded as strong evidence for purifying selection, but rather as a lack of evidence that purifying/background selection caused the previously observed signatures of convergent adaptation.

In lodgepole pine, a lack of clines in nucleotide diversity along environmental variables (Figs. 2a, 4a-e, Additional file 1: Figures S2a-e, S3a-e and Table S1) suggests that the intensity of purifying/background selection was either relatively spatially/environmentally uniform, varied inconsistently among the studied populations, or had weak enough effects as to be undetectable relative to the impact of other ecological/demographic processes. Population bottleneck events may cause the positive Tajima’s D values in lodgepole pine populations (Fig. 3) [25, 26]. Paleontological records indicate that lodgepole pine migrated progressively northward and founded small isolated populations following the last glacial maximum [27–30]. There is little spatial population genetic structure in lodgepole pine [31, 32], which likely explains the lack of clines observed in the current study. Another possible explanation for the positive Tajima’s D values is the genetic intraspecific admixture between varieties *Pinus contorta var. latifolia* and *var. contorta* (Fig. 6a). These two varieties appear to have extensive genetic connectivity, giving rise to an excess of common variants that would explain the positive Tajima's D values we observed [33]. Since the admixture levels are different within the studied populations (Fig. 6a), it is likely that the genetic diversity varied inconsistently, which can also result into the lack of clines presented in the current study.

Unlike lodgepole pine, interior spruce showed clines in nucleotide diversity along most of the studied environmental variables (Figs. 2b, 4f-j, Additional file 1: Figures S2f-j, S3f-j and Table S1). Clinal patterns of variation due to hybridization, selection and adaptation of the hybrid swarm have previously been found in the interior spruce complex [34–37]. In the present study, within the range of latitude 49° to 60°, the nucleotide diversity was lower in colder or northern range than that in warmer or southern range. These northern populations are primarily white spruce (Fig. 6b), in agreement with previous studies finding lower nucleotide diversity in white spruce populations [17] and high genetic diversity within admixed populations [34, 38]. It seems likely that hybridization between two species that differ in their levels of standing genetic variation is contributing to clinal nucleotide diversity in this species complex.

We found negative or near-zero values of Tajima’s D for spruce populations (Fig. 3). This is in line with the previous report that no recent bottleneck events could be inferred for the Engelmann spruce populations inhabiting British Columbia [38]. The clines and higher strength of correlations (*r*) in convergent genes than in non-convergent genes suggest geographic variation in the strength of linked selection (either positive or negative) may contribute to our observed patterns in the spruce complex, perhaps related to the studied environmental variables. However, there is insufficient evidence to confirm the role of spatially/environmentally varying purifying/background selection driving variation in nucleotide diversity, as most of our studied variables follow a generally coincident gradient with hybrid index. Thus, some combination of gene flow, local adaptation, and purifying selection likely causes the observed pattern in nucleotide diversity, but not in a way that lead to consistent patterns observed across both species. Previous studies have shown recombination decreases the effect of purifying selection on variation at linked neutral sites [6] and selection coefficient per base pair (selection density) is an important factor for inferring the strength and timing of selection against gene flow [39]. Future studies could take these factors into account to better demonstrate the complex relationship among divergent selection and gene flow.

## Conclusions

There are no consistent patterns in the correlations between nucleotide diversity and environmental variables in lodgepole pine and interior spruce. In spruce, we found stronger correlations between environmental variables and nucleotide diversity in convergent genes than that in non-convergent genes, but we did not see the same trend in pine. The clines in spruce may be related to spatially variable divergent or purifying/background selection, demography, or both processes. Overall, our results show that spatially/environmentally varying purifying/background selection is likely not the main driver of the convergent local adaptation patterns discovered previously [2]. Instead, positive and spatially divergent selection acting on the same genes in both species remains the most likely explanation for the previously observed convergent signatures. The mounting genomic data and annotated reference sequences of conifer species provide great opportunities to understand the genetic basis of local adaptation and the forces that drive evolution in forest tree genomes. We hope that this developing body of knowledge can lead to development of genomics tools that can be used to guide forest breeding practices in a changing climate.

## Methods

Lodgepole pine and interior spruce trees were sampled across heterogeneous environments in British Columbia and Alberta, Canada (Fig. 1). We obtained seeds of these sampled trees from the BC Ministry of Forests, Lands, and Natural Resource Operations Tree Seed Center (Surrey, BC, Canada). Seeds were stratified and grown in a growth chamber [40]. The sampling strategy maximized populations and minimized the number of individuals per population, so many populations did not have enough individuals to yield reasonable estimates of nucleotide diversity. We retained only populations with at least three sampled individuals, giving a total of 86 lodgepole pine and 50 interior spruce populations for the present study. For each population, 1961–1990 climate normals for four climatic variables were extracted using ClimateWNA [41], based on strong adaptation signals detected by Yeaman, Hodgins et al. [2]: Degree-days below 0 °C (DD_0), 30-year extreme minimum temperature (EMT), mean temperature of the coldest month (MCMT), and temperature difference between the warmest and coldest months (TD). In addition to the four climate variables, population latitude was also used in analyses.

The DNA sequences have been reported in the previous studies. Briefly, DNA was extracted from each individual and exome capture libraries were constructed and sequenced [40], then processed for SNP calling and population genomic analysis [2]. In the current study, we analyzed the population genetics parameters using the software ANGSD [42]. Nucleotide diversity was measured as the average number of pairwise differences across a given length of sequence (π). Nucleotide diversity and neutrality test statistics such as Tajima’s D were calculated for each population on a per-window basis, with a window size of 300 bp and a step size of 50 bp. For downstream analyses, we selected the non-overlapped windows according to the coordinates. We separated the non-overlapped windows to specific genic regions: whole genes, coding regions of genes (CDS), and non-coding regions of genes (non-CDS), using BEDTools [43]. The gene annotations for pine and spruce genomes were acquired from the previous study [2]. For each gene, nucleotide diversity and Tajima’s D were averaged across all windows belonging to each genic region (whole gene, CDS, non-CDS). Mean nucleotide diversity and mean Tajima’s D across all genes for each population were calculated and plotted against the environmental variables.

We separated genes into three categories based on the results of Yeaman, Hodgins et al. [2]: 1) convergent genes--- the 47 genes with convergent adaptation signatures; 2) non-convergent top candidate genes---genes associated with environmental variables in one or the other species, but without convergent adaptation signatures; and 3) background genes---genes without convergent local adaptation signatures and not associated with any environmental variable in either species. The latter two categories of genes were also combined and called non-convergent genes. The mean nucleotide diversities across all convergent genes, non-convergent top candidate genes, and background genes were regressed on environmental variables separately for different genic regions (whole gene, CDS, and non-CDS) over all populations within each species.

The strength of correlations (*r*) between environmental variables and nucleotide diversity of each gene were calculated separately for each gene and for the different genic regions across all populations within each species using the Pearson’s correlation coefficient. The *r* values of convergent genes and non-convergent genes were compared using a t-test within each species. The *r* values of convergent genes were compared between pine and spruce using the Pearson’s correlation coefficient.

To assess levels of admixture within individuals, SNPs were extracted from the non-coding regions in all individuals, using the SNP calling pipeline described by Yeaman, Hodgins et al. [2]. After filtering SNPs based on call rate (≥ 70%) and sequencing depth (≥ 7), admixture proportions were estimated using ADMIXTURE v1.23 [44], using default settings. For lodgepole pine, 217,567 SNPs were retained. Three reference *Pinus banksiana* and 6 reference *Pinus contorta var. contorta* samples, obtained and processed using the same methods described above, were included to capture any intra- or interspecific ancestry within the data. ADMIXTURE was run at K = 3 to separate the two species and to attempt to separate the two varieties present within lodgepole pine. For interior spruce, 190,397 SNPs were retained and no additional reference samples were used, as the dataset contains both parental species in addition to their hybrids. ADMIXTURE was run at K = 2 to separate the two parental species.

R and ggplot2 were used to analyze statistics and plot graphs [45, 46]. Maps were generated using R and ArcMap 10.6 [47].

## Additional file

Additional file 1: **Table S1.** Effects of environmental variables on nucleotide diversity. **Figure S1.** Clinal variation in nucleotide diversity in pine and spruce (LAT: latitude). **Figure S2.** Relationships between environmental variables and nucleotide diversity of coding-regions (CDS) in lodgepole pine and interior spruce for the convergent genes, non-convergent top candidate genes and background genes. **Figure S3.** Relationships between environmental variables and nucleotide diversity of non-coding-regions (non-CDS) in lodgepole pine and interior spruce for the convergent genes, non-convergent top candidate genes and background genes. **Figure S4.** Distribution of strength of correlations (*r*) between environmental variables and nucleotide diversity of coding-regions (CDS) in genes. **Figure S5.** Distribution of strength of correlations (*r*) between environmental variables and nucleotide diversity of non-coding regions (non-CDS) in genes. **Figure S6.** Correlations of strength of correlations (*r*) between environmental variables and nucleotide diversity of convergent genes between pine and spruce. **Figure S7.** Correlations of strength of correlations (*r*) between environmental variables and nucleotide diversity of coding-regions (CDS) of convergent genes between pine and spruce. **Figure S8.** Correlations of strength of correlations (*r*) between environmental variables and nucleotide diversity of non-coding regions (non-CDS) of convergent genes between pine and spruce. (DOCX 6371 kb)

## Data Availability

The sequence data analysed during the current study are available in the Short Read Archive (SRP071805; PRJNA251573).

## Abbreviations

CDS: Coding-regions
DD_0: Degree-days below 0 °C
EMT: 30-year extreme minimum temperature
LAT: Latitude
MCMT: Mean temperature of the coldest month
TD: Temperature difference between the warmest and coldest month
