# Supplementary material for: Purifying selection does not drive signatures of convergent local adaptation of lodgepole pine and interior spruce
Source: BMC Evol Biol. 2019 May 28;19:110. doi: 10.1186/s12862-019-1438-8 (PMC6537219; doi:10.1186/s12862-019-1438-8)
Supplement: Supplementary file 1 — Table S1. Effects of environmental variables on nucleotide diversity. Figure S1. Clinal variation in nucleotide diversity in pine and spruce (LAT: latitude). Figure S2. Relationships between environmental variables and nucleotide diversity of coding-regions (CDS) in lodgepole pine and interior spruce for the convergent genes, non-convergent top candidate genes and background genes. Figure S3. Relationships between environmental variables and nucleotide diversity of non-coding-regions (non-CDS) in lodgepole pine and interior spruce for the convergent genes, non-convergent top candidate genes and background genes. Figure S4. Distribution of strength of correlations (r) between environmental variables and nucleotide diversity of coding-regions (CDS) in genes. Figure S5. Distribution of strength of correlations (r) between environmental variables and nucleotide diversity of non-coding regions (non-CDS) in genes. Figure S6. Correlations of strength of correlations (r) between environmental variables and nucleotide diversity of convergent genes between pine and spruce. Figure S7. Correlations of strength of correlations (r) between environmental variables and nucleotide diversity of coding-regions (CDS) of convergent genes between pine and spruce. Figure S8. Correlations of strength of correlations (r) between environmental variables and nucleotide diversity of non-coding regions (non-CDS) of convergent genes between pine and spruce. (DOCX 6371 kb) [file 12862_2019_1438_MOESM1_ESM.docx]

**Additional file 1: Table S1** Effects of environmental variables on nucleotide diversity

| Environmental variables* | Genic regions† | Pine | | | Spruce | | |
| --- | --- | --- | --- | --- | --- | --- | --- |
|  |  | On convergent genes | On non-convergent top candidate genes | On background genes | On convergent genes | On non-convergent top candidate genes | On background genes |
| DD_0  (degree-days) | Gene | 0.00  (0.55) | 0.00  (0.57) | 0.01  (0.51) | 0.20  (0.00) | 0.19  (0.00) | 0.07  (0.06) |
|  | CDS | 0.00  (0.75) | 0.01  (0.54) | 0.01  (0.52) | 0.20  (0.00) | 0.18  (0.00) | 0.08  (0.05) |
|  | Non-CDS | 0.00  (0.62) | 0.00  (0.55) | 0.01  (0.51) | 0.23  (<0.00) | 0.19  (0.00) | 0.08  (0.05) |
| EMT  (℃) | Gene | 0.01  (0.49) | 0.01  (0.32) | 0.00  (0.64) | 0.05  (0.14) | 0.05  (0.13) | <0.00  (0.55) |
|  | CDS | 0.00  (0.73) | 0.01  (0.31) | 0.00  (0.64) | 0.06  (0.10) | 0.05  (0.14) | 0.01  (0.52) |
|  | Non-CDS | 0.00  (0.59) | 0.01  (0.31) | 0.00  (0.64) | 0.06  (0.10) | 0.05  (0.13) | 0.01  (0.54) |
| LAT  (∘N) | Gene | 0.01  (0.36) | 0.01  (0.30) | 0.02  (0.24) | 0.16  (0.00) | 0.15  (0.01) | 0.05  (0.11) |
|  | CDS | 0.00  (0.68) | 0.02  (0.24) | 0.02  (0.23) | 0.15  (0.01) | 0.15  (0.01) | 0.05  (0.05) |
|  | Non-CDS | 0.01  (0.48) | 0.01  (0.35) | 0.02  (0.24) | 0.19  (0.00) | 0.16  (0.00) | 0.05  (0.10) |
| MCMT  (℃) | Gene | 0.00  (0.56) | 0.00  (0.64) | 0.00  (0.64) | 0.25  (<0.00) | 0.26  (<0.00) | 0.09  (0.04) |
|  | CDS | 0.00  (0.75) | 0.00  (0.62) | 0.00  (0.64) | 0.23  (<0.00) | 0.26  (<0.00) | 0.09  (0.04) |
|  | Non-CDS | 0.00  (0.64) | 0.00  (0.63) | 0.00  (0.64) | 0.29  (<0.00) | 0.26  (<0.00) | 0.09  (0.03) |
| TD  (℃) | Gene | 0.00  (0.60) | 0.01  (0.35) | 0.00  (0.79) | 0.38  (<0.00) | 0.36  (<0.00) | 0.14  (0.01) |
|  | CDS | 0.00  (0.68) | 0.01  (0.36) | 0.00  (0.81) | 0.33  (<0.00) | 0.35  (<0.00) | 0.13  (0.01) |
|  | Non-CDS | 0.00  (0.63) | 0.01  (0.39) | 0.00  (0.78) | 0.42  (<0.00) | 0.37  (<0.00) | 0.14  (0.01) |

Upper numbers are *r^2^* values for linear regression; lower numbers within parentheses are the *p*-values for linear regression. *DD_0: degree-days below 0 ℃; EMT: 30-year extreme minimum temperature; LAT: latitude; MCMT: mean temperature of the coldest-month; TD: temperature difference between the warmest and coldest months. †CDS: coding-regions; non-CDS: non-coding-regions

**Additional file 1: Figure S1** Clinal variation in nucleotide diversity in pine and spruce (LAT: latitude). Clinal variation in nucleotide diversity of coding-regions (CDS) in pine (a) and spruce (b). Clinal variation in nucleotide diversity of non-CDS in pine (c) and spruce (d)

**Additional file 1: Figure S2** Relationships between environmental variables and nucleotide diversity of coding-regions (CDS) in lodgepole pine and interior spruce for the convergent genes, non-convergent top candidate genes and background genes. Panels show the comparison of relationships between nucleotide diversity and: (a, f) degree-days below 0 ℃ (DD_0); (b, g) 30-year extreme minimum temperature (EMT); (c, h) latitude (LAT); (d, i) mean temperature of the coldest-month (MCMT); (e, j) temperature difference between the warmest and coldest months (TD)

**Additional file 1: Figure S3** Relationships between environmental variables and nucleotide diversity of non-coding-regions (non-CDS) in lodgepole pine and interior spruce for the convergent genes, non-convergent top candidate genes and background genes. Panels show the comparison of relationships between nucleotide diversity and: (a, f) degree-days below 0 ℃ (DD_0); (b, g) 30-year extreme minimum temperature (EMT); (c, h) latitude (LAT); (d, i) mean temperature of the coldest-month (MCMT); (e, j) temperature difference between the warmest and coldest months (TD)

**Additional file 1: Figure S4** Distribution of strength of correlations (*r*) between environmental variables and nucleotide diversity of coding-regions (CDS) in genes. Grey polygons show the density of *r* values of CDS in all genes. Red bars on the x-axis show the *r* values of CDS in convergent genes. Panels show distribution of *r* values for correlations between nucleotide diversity and: (a, f) degree-days below 0 ℃ (DD_0); (b, g) 30-year extreme minimum temperature (EMT); (c, h) latitude (LAT); (d, i) mean temperature of the coldest-month (MCMT); (e, j) temperature difference between the warmest and coldest months (TD)

**Additional file 1: Figure S5** Distribution of strength of correlations (*r*) between environmental variables and nucleotide diversity of non-coding regions (non-CDS) in genes. Grey polygons show the density of *r* values of non-CDS in all genes. Red bars on the x-axis show the *r* values of non-CDS in convergent genes. Panels show distribution of *r* values for correlations between nucleotide diversity and: (a, f) degree-days below 0 ℃ (DD_0); (b, g) 30-year extreme minimum temperature (EMT); (c, h) latitude (LAT); (d, i) mean temperature of the coldest-month (MCMT); (e, j) temperature difference between the warmest and coldest months (TD)

**Additional file 1: Figure S6** Correlations of strength of correlations (*r*) between environmental variables and nucleotide diversity of convergent genes between pine and spruce. (a) Correlations of *r* values between degree-days below 0 ℃ (DD_0) and nucleotide diversity of convergent genes between pine and spruce (*r* = -0.30, *p*-value = 0.04); (b) Correlations of *r* values between 30-year extreme minimum temperature (EMT) and nucleotide diversity of convergent genes between pine and spruce (*r* = -0.32, *p*-value = 0.03); (c) Correlations of *r* values between latitude (LAT) and nucleotide diversity of convergent genes between pine and spruce (*r* = -0.18, *p*-value = 0.22); (d) Correlations of *r* values between mean temperature of the coldest-month (MCMT) and nucleotide diversity of convergent genes between pine and spruce (*r* = -0.33, *p*-value = 0.02); (e) Correlations of *r* values between temperature difference between the warmest and coldest months (TD) and nucleotide diversity of convergent genes between pine and spruce (*r* = -0.34, *p*-value = 0.02)

**Additional file 1: Figure S7** Correlations of strength of correlations (*r*) between environmental variables and nucleotide diversity of coding-regions (CDS) of convergent genes between pine and spruce. (a) Correlations of *r* values between degree-days below 0 ℃ (DD_0) and nucleotide diversity of CDS of convergent genes between pine and spruce (*r* = -0.42, *p*-value < 0.00). (b) Correlations of *r* values between 30-year extreme minimum temperature (EMT) and nucleotide diversity of CDS of convergent genes between pine and spruce (*r* = -0.37, *p*-value = 0.01). (c) Correlations of *r* values between latitude (LAT) and nucleotide diversity of CDS of convergent genes between pine and spruce (*r* = -0.17, *p*-value = 0.24). (d) Correlations of *r* values between mean temperature of the coldest-month (MCMT) and nucleotide diversity of CDS of convergent genes between pine and spruce (*r* = -0.43, *p*-value < 0.00). (e) Correlations of *r* values between temperature difference between the warmest and coldest months (TD) and nucleotide diversity of CDS of convergent genes between pine and spruce (*r* = -0.37, *p*-value = 0.01)

**Additional file 1: Figure S8** Correlations of strength of correlations (*r*) between environmental variables and nucleotide diversity of non-coding regions (non-CDS) of convergent genes between pine and spruce. (a) Correlations of *r* values between degree-days below 0 ℃ (DD_0) and nucleotide diversity of non-CDS of convergent genes between pine and spruce (*r* = -0.32, *p*-value = 0.03). (b) Correlations of *r* values between 30-year extreme minimum temperature (EMT) and nucleotide diversity of non-CDS of convergent genes between pine and spruce (*r* = -0.35, *p*-value = 0.01). (c) Correlations of *r* values between latitude (LAT) and nucleotide diversity of non-CDS of convergent genes between pine and spruce (*r* = -0.13, *p*-value =0.37). (d) Correlations of *r* values between mean temperature of the coldest-month (MCMT) and nucleotide diversity of non-CDS of convergent genes between pine and spruce (*r* = -0.35, *p*-value = 0.02). (e) Correlations of *r* values between temperature difference between the warmest and coldest months (TD) and nucleotide diversity of non-CDS of convergent genes between pine and spruce (*r* = -0.33, *p*-value = 0.02)
